# Retrospect of the Medical Sciences

**Published:** 1843-08-05

**Authors:** 


*Case of Popliteal Aneurism cured by Compression of the Femoral Artery.* By EDWARD HUTTON, M.D. Surgeon to the Richmond Hospital.

Michael Duncan, æt. 30, a labourer of rather healthy appearance, but of intemperate habits, was admitted into the Richmond Hospital on the 3d of October, 1842. He stated that ten days previously, while suffering from cramp in the right leg, to which he had been subject for the last year, he, for the first time, discovered a tumour in his right ham, which was then equal in size to a hen's egg ; in three days afterwards he observed some swelling in the foot and ankle, and felt pain along the outside of the leg. At the time of his admission into the hospital the tumour had somewhat increased in size, and was found to occupy the lower part of the popliteal space. It pulsated strongly, and when the femoral artery was compressed in the groin the tumor admitted of considerable collapse. The compression being removed, it again became extended, and the "purring thrill" attended the re-entrance of the blood into the aneurismal sac. The leg was somewhat swollen and its veins turgid, and he complained of prickling sensations in the limb. His pulse was 60 and regular, and his general health appeared unaffected. The nature of his case was explained to him, and the operation of tying the femoral artery proposed. To this he declined to submit in the first instance, and expressed a desire that other means might be tried. For three or four weeks he maintained the horizontal posture, and a compress and bandage was applied ; but as the tumor gradually increased in size, and as he suffered pain from the pressure, this treatment was discontinued.

Nov. 1st.—The patient being still reluctant to undergo the operation, I resolved to try compression of the femoral artery, and I entertained some hope of success from being informed by Mr. Adams that the late Mr. Todd had succeeded in a similar case, of which no account has been published. Having at hand an instrument constructed for the suppression of secondary hæmorrhage, after ligature of the femoral artery, I applied it in this case. It was so contrived as to admit of pressure being made by a screw and pad upon the course of the femoral artery, and the counter-pressure upon the opposite surface of the limb, without interfering with the collateral circulation.

In the first instance the compression was made upon the femoral artery in the middle third of the thigh, and although it was effectual in compressing this vessel, it produced so much uneasiness that it could not be sustained, and after a few applications the apparatus was removed, and adapted to the upper part of the limb.

12th.—The femoral artery was compressed as it passes from the pelvis under Poupart's ligament, and the pressure maintained for more than four hours.

14th.—The tumor feels rather more solid ; the purring thrill, before felt on the re-entrance of the blood into the sac, is no longer sensible ; the pulsation as before.

18th.—No change in the tumor.

19th.—The circumference of the limb at the seat of the tumor is a quarter of an inch less than at the last measurement.

22d.—Duration of compression three hours ; the pulsation returned after its removal.

24th.—Artery compressed six hours ; same result.

25th.—He was unable to bear the application from soreness in the groin ; he complained also of some pain in the tumor.

26th.—The compression was resumed, and continued for four hours ; when the instrument was removed, the pulsation had ceased in the tumor, which felt solid, and was free from pain.

27th.—The pulsation had, in a slight degree, returned ; compression applied for six hours.

28th.—No pulsation was now felt in the tumor. It had decreased in size, and was solid.

29th.—The compression was maintained six hours; no pulsation can be felt; compression applied for three hours.

Dec. 1st.—An artery, about the size of the temporal, is felt pulsating along the surface of the tumor, which is quite solid, much diminished in size, and is altogether free from pulsation. The use of the instrument was now discontinued. The femoral artery pulsates naturally.

On the 7th of December the temperature of the legs was examined at the calf.

Temperature of the aneurismal limb, 86° Fahrenheit; of sound limb, 90°.

| | | Aneur. | Sound limb. |
|---|---|---|---|
| Dec. 12th.—Temperature | | 88° | 90° |
| " 20th. | " | 90° | 91° |
| " 21st. | " | 91° | 91° |

On the 27th of December the tumor was reduced to the size of a small walnut, and felt very hard. He was this day discharged at his own request.

In six weeks he visited the hospital at my request. The tumor was about the size of a nutmeg, and solid. He had been at his usual employment.

*Remarks.*—Since this case occurred Dr. Cussack has treated with success, by similar means, a case of popliteal aneurism in Dr. Stevens' Hospital, and Dr. Bellingham another in St. Vincent's Hospital. It would appear that this plan of treatment has been too hastily abandoned by the profession, probably from the compression employed being so excessive as to render it quite insupportable to the patient. The least possible pressure which may be sufficient to close the vessel should be used, and when this cannot be sustained, it will prove of use to partially compress the artery, so as to lessen the impulse of the circulation. In cases where the aneurismal diathesis exists, this treatment would seem to be demanded before recourse should be had to an operation.—*Lond. Med. Gaz.*

---

DR. FRANCIS WILLIS, in a second edition of his "Treatise on Mental Derangement," states that in most recent cases of the disease, even the most outrageous, the patient requires *support* and not *reduction* of nutrition, the malady being caused, as he considers, by a weakness, not an over-tension of the nerves. He, however, advocates personal restraint, as both safe and merciful. He subdivides unsoundness of mind into three degrees; delirium, in which incoherent images and impressions pass uncontrollably over the mind ; insanity, where the mind, though awake to present objects, is in a distorted position, from which it refuses to be moved; and derangement of mind—between the other two, though with difficulty distinguishable from the latter. He says little on the subject of monomania, and the responsibility attendant on the acts of those who are its supposed victims, but on this subject the following remarks have been made by a commentator: Where we find that a disposition, originally suspicious, obstinate, reckless, and blood-thirsty, has from indulgence of its propensities, darkened down into the twilight of insanity, and incited a man to outrage and crime, we ought to be cautious how we allow the belief that the urgency of his passions had arisen to disease so as to *exculpate* him from all guilt. A boy was tried for the murder of a companion before a French tribunal. Having been indulged by his mother, from childhood, in every bad passion, he had become, at last, totally unable to restrain himself from acts of violence, and was acquitted on the score of insanity—a dangerous and unsound decision. Considering such "morbid impulse" as only an exasperation of the natural disposition, should not a prisoner thus placed be held amenable to society for a crime committed remotely, though not immediately, in consequence of *responsible* depravity. For a criminal who has, by indulgence, reduced himself to commit crime, is, in a manner, during his admitted sanity, accessory to his own crime; and although, in giving way to his passions, it was not his intention that they should result in acts of atrocity, yet, acting on the provisions of the criminal law, we may view the homicidal monomaniac as *a separate individual, prompted by his former self*, and, even admitting the absence of intention, justly to be held liable to the criminal law. Blackstone lays down that he who in any wise commands or counsels another to do an unlawful act, is accessory to all that ensues thereupon. Therefore the monomaniac, become criminal from indulgence, is an *accessory before the fact* to a crime afterwards perpetrated by himself—guilty in an equal degree with his principal, and punishable alike! This, however, is granting the supposition that he has lost the power of control over himself. But in an eloquent lecture at the Royal Institution on the 26th ult. the lecturer advanced that it was very rarely that in the insane the brain was found to be diseased in *every* part; and he adduced numerous instances to prove that in a majority of cases the patient was capable of exercising a control over his disposition, by abandoning the exercise of an organ; or a portion of the brain, that was phrenologically supposed to be unsound, for that of one which was, phrenologically, presumed to be sound.

*Lancet.*

### BLACK CATARACT.

At the Academy of Sciences, Paris, May 29, 1843. M. Magne forwarded the following case of this rare and curious disease:—

A female, above sixty years of age, had laboured under some affection of the eyes, for which she had consulted a great number of oculists. She was quite blind; the eyeballs were prominent; the sclerotica appeared to be thin; the iris well shaped, but perfectly immoveable; bottom of the pupil dark, as in the healthy state.

From these and other symptoms, the disease was supposed to be amaurosis; but a second examination of the patient was made in a darkened chamber, and with the aid of a candle, as recommended by M. Sanson. The deep-seated images were absent, and the author accordingly declared the case to be one of black cataract, with adhesion of the iris. The diagnosis having been confirmed by M. Cruveilhier, the lens on the right side was depressed, on the 25th of March, 1843. The adhesions of the iris were numerous; but as soon as the capsule was lacerated, the dark colour of the lens became evident, and, on depressing it, several black fragments were detached.

On the second day after the operation the pupil appeared to be less contracted, the base being quite dark, but on the following day it was closed by a white substance. M. Cruveilhier regarded this as the lens, which had come forwards, after having lost its dark colour in the vitreous humor. The operation was unsuccessful, and was, therefore, repeated in a fortnight; but the first touch of the needle showed that the body supposed to be the lens was, in reality, the capsule, which was extremely soft and elastic. A few shreds were removed with much difficulty, and the patient recovered but a very imperfect power of vision.—*Prov. Med. Journ.*

### STATISTICS OF ANAL FISTULA.

During the five years from 1836 to 1840 inclusive, 119 patients were operated on at the Hôtel Dieu, Paris, for fistula in ano. Of these persons 110 left the hospital cured, and 9 (or 1 in 12) died. The mortality from the operation was progressively less in proportion from the first to the last-mentioned year. Of the 119 individuals operated on, 32 were of ages between 15 and 25 (4 only being under 20 years of age,) 55 from 25 to 40, and 32 between 40 and 60 years old (only 3 being more than 51 years old.) Only 12 of the whole 119 were females. Sedentary occupations, and whatever is productive of habitual constipation, have been considered fruitful causes of fistula; but the evidence elicited from the individuals suffering from the disease was by no means corroborative of such statements. The patients included indifferently sawyers, carpenters, masons, bakers, porters, and other persons accustomed to perpetual exercise, as well as tailors, bootmakers, cutlers, cabinet-makers, and others employed in sedentary pursuits. Some connection of fistula with a tuberculous diathesis seemed, however, to be apparent.—*Lancet, from Gaz. Med. de Paris.*

### CONSERVATION OF MISTURA FERRI COMPOSITA.

Mr. Strutton, in the "Chemical Gazette," gives the following formula for the mistura ferri composita, by which its decomposition may be prevented:—

Myrrh, two drachms;
Carbonate of potash, one drachm;
Rose water, fifteen and a half ounces;
Spirits of nutmeg, an ounce;
Sugar, two ounces. Mix according to the Pharmacopœia and dissolve.

Sulphate of iron, two and a half scruples, dissolved in two and a half ounces of rose water.

When required, add to seven drachms of the first mixture, one drachm of the latter, which saves the trouble of preparing it for every prescription, and is equal to the mixture being fresh made every time it is wanted.—*Prov. Med. Journ.*

### IODURATED SYRUP OF SARSAPARILLA.

M. Ricord is in the habit of employing the following formula in the treatment of tertiary syphilis:—

Syrup of sarsaparilla, five hundred scruples;
Hydriodate of potass, sixteen scruples.

He commences with three teaspoonfuls a-day (morning, noon, and night,) and gradually carries the dose to twelve spoonfuls. Each dose is given in a glassful of the decoction of saponaria, hops, &c.
*Ibid, from Gaz. des Hopitaux.*

QUININE IN TRAUMATIC FEVER.

A case has lately occurred in the Hôpital des Enfans at Paris, in which M. Guersant has employed, with entire success, the sulphate of quinine in the treatment after amputation of both lower extremities. The patient, a poor boy, whose limbs had been frozen by exposure during a winter's night, and afterwards most injudiciously bathed in hot water, was brought to the hospital, the limb exhibiting a discoloured spotty appearance, and other symptoms of incipient gangrene. This condition in six days, notwithstanding the application of bark-poultices and bottles of hot water to the extremities, so greatly increased as to render necessary an immediate amputation of both legs. The operation was successfully performed, and with little suffering to the patient, who gradually recovered, though not without indications of considerable fever and erysipelatous inflammation, both of which were subdued by doses of sulphate of quinine, amounting to twelve grains in a day. M. Guersant cites other cases, also, in which he has employed the same medicine with success, in all of which the leading feature was purulent reabsorption.—*London Lancet, from Gazette des Hôpitaux, March* 14.

PATHOLOGY AND TREATMENT OF RICKETS AND MOLLITIES OSSIUM.

The diseases which exert the greatest influence over the condition of the bones, altering them from their normal state, are scrofulous affections, to which rickets and mollities ossium may be considered as belonging. In these two diseases, the earthy matter of the bone becomes diminished, and the bone falls into a state such as if it had been macerated in muriatic acid; supple, flexible, and ill adapted to serve as a support to the other organs of the body. The cartilage itself submits to an essential alteration, and is incapable of being converted by boiling into gelatine.

Concurrently with these changes, phosphate of lime is eliminated in large quantities with the urine. This salt, otherwise little soluble, and discharged generally only in small quantity by the kidneys, is according to Berzelius, readily soluble in lactic acid; anything, therefore, which causes a superabundance of this acid in the system is capable of depriving the organism of a large share of the earthy matter of the bones. Sugar of milk, grape sugar, starch, and gum, are readily converted into lactic acid, but they are so in the stomach only when digestion is ill-performed, in which case lactic acid may be an abundant product in the system.

Rickets and mollities ossium, therefore, are not essentially diseases of the bones, but seem to be results of imperfect digestion or nutrition; to improve which is consequently our first indication. None of the substances readily converted into lactic acid should be taken, as sugar, starch, gum, &c., nor even milk (rickets are often the consequence of children having been too long suckled,) but animal food and such other as is freely digestible should be chosen, in aid of which we ought to employ such medicines as may restore the general tone of the system.—*Lancet, from Marchand, in Journ. de Pharm. et de Chimie.*

*On the Characters and Structural Peculiarities of a group of Morbid Growths in which cancerous affections are included.* By DR. HODGKIN.

This paper was in continuation of a subject which had already been brought before the Royal Medical and Chirurgical Society on a former occasion by the same writer.

After describing the different appearances revealed by the improved microscopes of the present day, the author endeavored to connect the nucleated cells, which Miller has shewn to exist in these structures, with the production of those compound cysts which were described in the former paper, and pointed out as affording the type of the adventitious structures referred to.

The following are the conclusions which the author is desirous of drawing from the observations contained in the paper:—

1st. The unrestricted confirmation of the views contained in the former paper as to the existence of the type of compound serous cysts in the adventitious structures referred to. The author had not only found it in man, but in several of the inferior species of mammalia, and in birds. Several able observers had, on examination, coincided with his views, and he mentioned the late Professor Delpech, and the present Professor Rokitanski, having personally informed him of their having, independently, been induced to adopt his views.

2d. That the microscopic examination of these tissues, though extremely interesting, does not furnish perfectly conclusive tests of any particular form of adventitious structure to which a specimen may belong, but that it demonstrates the application of the nucleated cell theory, whilst it is fatal to that of cancerous matter being formed in the blood and eliminated at the spots at which the tumors become manifest. It therefore furnishes an important argument in favor of operation, though other practical considerations require to be attended to before operation is decided on.

3rd. That to have a complete view of the mode of production of these structures, we must combine the cell theory of Schwann and Müller, the coagulation principle which the author had previously suggested, and the process of organization investigated by Mr. Kiernan—three stages of development which appear to occur in the order just enumerated; and that none of the phenomena, taken singly, is an adequate test of malignancy, which, as stated in his first paper, must be regarded as the sum of several characters.

4th. That chemical analysis, though extremely important and interesting, affords an imperfect and inadequate criterion, as the principles concerned may vary or be changed in the progress of development.

5th. That, in operating for the removal of a tumor of this class, it is extremely important to leave behind none of those minute cysts which often form granules in the surrounding cellular membrane, though it may appear in other respects perfectly healthy. This appears to be a mode of extension of the disease independent of inflammation.

6th. That experience teaches us that the infiltrated form of these diseases occur in the structures in the neighborhood of the purely adventitious growth, when these structures have been the seat of inflammation, and that the chances of success from operation are consequently infinitely diminished when such surrounding inflammation has taken place. The presence of the peculiar matter of the disease in the interior of the vessels appears to be one of the modes in which infiltration, the result of inflammation, exhibits itself, and is, therefore, not a valid argument in favor of the pre-existence of such matter in the circulating blood.—*London Medical Gazette*, June 23, 1843.

---

NEW LIMITS FOR THE PERIOD OF LACTATION.

Dr. Loudon, in a recently published work on population, has named the period of three years as that during which the infant should receive its nourishment by lactation. His argument is the following: "Of all mammiferæ, none is so long as man in being able to sustain itself upon its legs, in reaching the age of puberty and adult age, and, taking his size into consideration, none is endowed with a longevity comparable with his. Man is completely developed at 21; few men have lived till 150. Now, if we divide the first number by 7, and the second by 49, we shall find that the period of lactation for the human race ought to be three years."—*Provin. Med. Journ.* June 24, 1843.

---

DIAGNOSTIC SIGN OF DISLOCATION OF THE THIGH-BONE INTO THE ISCHIATIC NOTCH.

Mr. Syme has recently narrated, in the "London and Edinburgh Monthly Journal," a case where the occurrence of the dislocation was determined by the presence of a particular sign, which appears to be of much importance in the diagnosis of a dislocation which Sir Astley Cooper has described as "the most difficult both to detect and reduce." There is less deformity and fixture of the limb than in any other of the displacements of the thigh-bone. "This obscurity (says Mr. Syme) is much increased by attempts to effect reduction, since a moderate degree of extension almost entirely removes the shortening and inversion, which are usually considered the most characteristic symptoms. I think it, therefore, of consequence to state, that there is another feature of the injury which, according to my experience, is never absent—always well marked—and not met with in any other injury of the hip-joint, whether dislocation, fracture, or bruise. This is an arched form of the lumbar part of the spine, which cannot be straightened so long as the thigh is straight, or in a line with the patient's trunk. When the limb is raised or bent upwards upon the pelvis, the back rests flat upon the bed; but so soon as the limb is allowed to descend, the back becomes arched as before. By attention to this symptom, I have been enabled to recognise the existence of dislocation into the ischiatic notch, when it had been unnoticed by others; and, on one occasion, when it was supposed that the replacement had been effected through powerful extension by the pulleys."—*Provin. Med. Journ.* June 24, 1843.

---

UREA DISCOVERED IN THE BLOOD DURING THE EXISTENCE OF PULMONIC INFLAMMATION.

Dr. F. Simon, having taken the coagula afforded by six bleedings performed upon males and females laboring under inflammation of the lungs, with a view of procuring a quantity of hæmato-globuline, found that, by the action of alcohol, &c., and, finally, the addition of nitric acid to the alcoholic extract, and desiccation under the air-pump, he procured a crop of crystals, which, under the microscope, presented themselves as an aggregate of rhombic tables, and which he discovered to be the nitrate of urea.—*Ibid, from Müller's Archiv.*

STATISTICS OF CANCER.

The following are results of researches on the prevalence of this disease throughout France, which have been made with much care and accuracy on the part of M. Le Roy d'Etiolles:—

Of 2781 cases occurring in the practice of 174 surgeons, 1227 happened in individuals above forty, and 1061 to others above sixty years of age. The cases of cancer of the uterus were about thirty per cent.; of the breast twenty-four per cent. Cancer of the mouth was in women only as one to one and a half per cent., while in men (probably from the use of the tobacco-pipe) it was as much as twenty-six per cent. Cancers supposed to have been of hereditary transmission figured only as 1 in 278 (?); while those induced by scrofula were as 1 in 10; and by syphilis as 1 in 5.

The most useful part of the inquiry is that which is brought to bear upon the utility or otherwise of operating on cancers. Out of 1172 patients not operated on, 18 lived for more than thirty years after the first appearance of the disease; while out of 801 operated on by excision or caustic, the existence of only 4 was prolonged for a similar lapse of time; 14 patients operated on, and 34 not operated on, lived for a period of from twenty to thirty years; and 88 in the first category, and 228 in the second, lived from six to twenty years after the first appearance of the disease. The ordinary duration of life after this period among persons not operated on, is said to be five years for men, and five and a half for women; while among those operated on, it is no more than five years and two months for men, and six years for women.

From these results the natural conclusion is that the ablation of cancer (leaving out of account the risks attending the operation itself) does little, even when successful, to prolong life, and is therefore (in France, at least) of very questionable utility. Results like these, startling as they may seem, and however they may demand subsequent corroboration, are, at least, indications of the light which statistical science is enabled to throw upon the actual and relative value of many of the aids in medicine and surgery, of which we at present avail ourselves.—*Ibid.*

---

INFLUENCE OF THE SEASONS ON DISEASES AND MORTALITY.

Dr. W. A. Guy, of King's College, has recently taken considerable pains to ascertain the extent of influence which the *seasons* and the *weather*, in this country, especially in London, exercise in the production of the aggravation of disease and mortality, and in the May number of the "Journal of the Statistical Society of London," has published the facts bearing on the subject, which he has collected. The deductions which he makes from these are, "that there is no relation, whether direct or inverse, between the mortality and any single condition of the air, but that the sickness follows the exact order of the temperature and dew-point, varying directly as each of them.

"That the disorders which determine the order of the quarters in respect of sickness, or which may be said to govern the law of sickness, are the febrile affections, catarrh, the contagious exanthemata, and disorders of the digestive organs; to which may be added scrofula, gout, dropsy, &c. These prevail chiefly in the third and first quarters of the year. The diseases of the organs of respiration follow precisely the inverse order of those already named, being most prevalent in the second and fourth quarters; and these are the diseases which chiefly govern the order of mortality.

"As a general rule, but one admitting of many exceptions, it may be stated, that the amount of sickness tends to vary directly, and the amount of mortality inversely, as the temperature.

"No class of diseases has been found to follow the order of the total mortality; in other words, there was no class of diseases that by the great difference in the mortality which it occasioned in the several quarters of the year might be said to determine or govern the total mortality. The deaths, however, from diseases of the organs of respiration and from old age followed the order of the total mortality, except that the second and third quarters were inverted.

"Were it not for the excessive mortality during the summer months, the deaths from diseases of the chest, coupled with those from old age, would have governed the mortality; and it is highly probable that if a severe winter were to coincide with a mild summer, this would prove to be the case.

"It would appear that on an average of a number of years the mortality for the metropolis corresponds with that for the entire kingdom, inasmuch as the first quarter is the most fatal, and the third the least fatal, but that the place of the second and fourth quarters is inverted, the autumn being most fatal in the metropolis, and the spring in the country generally."—*Ibid.*

---

### PARALYSIS OF THE BLADDER CURED BY THE TINCTURE OF CANTHARIDES.

A patient was lately admitted into the Hôpital de la Pitie with paralysis of the bladder, for the relief of which all ordinary methods of treatment had failed. M. Lisfranc ordered the direct application of tincture of cantharides to the bladder by the following mode: One drop of the tincture was let into the organ through a catheter, and followed by an injection of simple lukewarm water. Next day two drops were similarly instilled, and the like operation was repeated night and morning for several succeeding days, an additional drop of the tincture being added on each successive occasion. By this method of treatment a cure was soon effected. M. Lisfranc found no perceptible local irritation to result from the use of the tincture in an undiluted form, while the direct application of the remedy to the organ affected was clearly preferable, in every respect, to its internal administration.—*Ibid.*

---

### ANATOMY OF THE GANGLIONIC NERVES.

The researches of Volkmann and Bidder have confirmed—what, indeed, the march of science had previously caused to be little doubted by physiologists—that the ganglionic or sympathetic is not a mere offset from the cerebro-spinal nervous system, but an independent system of itself. The above anatomists have, by the aid of the microscope, verified a great difference in the arrangement of the nervous fibrillæ in the two systems. The fibrillæ of the sympathetic nerves are distinguished from those of the spinal cord, by being paler, thinner, and containing less granular matter. Collected in bundles they have a greyish-yellow tinge. Where they communicate with the spinal nerves, the fibrils of each system of nerves may be distinctly traced by the aid of the microscope. Those of the sympathetic system are seen not only to penetrate to the centre of the spinal nerves, but to spread themselves around the circumference of the latter, where, by a careful measurement, the greater number are found to be distributed. If the sympathetic nerves originated from those of the spinal cord, say Volkmann and Bidder, we ought to find fibrils belonging to them in the roots of the spinal nerves. Now, if these roots be examined, scarcely one *sympathetic* among fifty medullary fibrils will be found; though they ought in such a case to be met with there in greatest number. The sympathetic nerves in reality originate in the ganglia; but not only the ganglia of the sympathetic cord, but those also on the posterior branches of the spinal nerves. These latter ganglia especially give origin to the sympathetic filaments destined to unite with the posterior ramifications of the spinal nerves, a fact which gives probability to the hypothesis of Weber respecting the use of the spinal ganglia.—*Ibid, from Froriep's Notizen.*

---

### USE OF ELDER-BARK IN CHRONIC DROPSIES.

The decoction and extract of this vegetable substance are reported to be remarkably efficacious as hydragogues, producing so speedy an effect on the urinary and fæcal secretions as to make it needless to use more than two or three applications. The proportions for the decoction consist of a couple of handfuls of the bark to a quart of water: dose, a wine-glassful a day. The extract is administered in France in the form of pills, of one and a half grain each, of which from six to ten are taken in the twenty-four hours.—*Ibid, from Journ. de Med. et de Chir. Pratique.*

---

Dr. Frampton has just made public a new and elegant method of testing the presence of corrosive sublimate, grounded on the known affinity of silver for mercury. He added a grain of bichloride of mercury to four ounces of tea with sugar and milk; then boiled this mixture with salver-dust, and poured off the supernatant fluid. The residue was boiled with liquor potassa for the removal of organic matters; and liquor ammoniæ was next added to dissolve the chloride of silver formed. The sediment was now washed and dried, and on placing it in the bulb of a tube and applying heat, a ring of globules of metallic mercury collected around the neck of the tube. Dr. Frampton procured similar results by operating in the same manner on mixtures of the bichloride with a solution of gelatine, and with sanguinolent serum from a hydrocephalic subject; but in these cases muriatic acid was employed to acidulate the mixture before the addition of the silver. The silver should for convenience be moistened with a little distilled water before being added to the mixture.—*Ibid.*

---

### TEST FOR IODINE IN MINERAL WATERS.

M. Boujean, of Chamberg, affirms that nitric acid is the most effective test for iodine, when contained in a mineral water. It is twenty times as delicate as chlorine. With the aid of nitric acid, M. Boujean discovered iodine in the sulphur springs of Chevillard, near Aix, in which its existence was not discoverable by any other means, and also in aqueous solutions of sponge, lichen islandicus, fucus, &c., after having cleared the solution by means of charcoal.—*Provin. Med. Jour.*

